# The Relationship Between Severity of Apical Periodontitis and Next‐Generation Systemic Inflammatory Biomarkers

**DOI:** 10.1111/aej.70078

**Published:** 2026-04-09

**Authors:** Gözde Akbal Dincer, Rezan Sungur Güzel

**Affiliations:** ^1^ Faculty of Dentistry, Department of Endodontics Dentistry Istanbul Okan University Istanbul Turkey

**Keywords:** haematological biomarkers, neutrophil‐to‐lymphocyte ratio, periapical index, platelet inflammatory value, systemic immune‐inflammation index

## Abstract

Apical periodontitis (AP) is an endodontic infection that may persist asymptomatically and be reflected in systemic inflammatory status. This study aimed to evaluate the association between radiographic severity of AP and next‐generation systemic inflammatory biomarkers in systemically healthy adults. Archived clinical, radiographic and haematological data of 206 adult patients treated under general anaesthesia between 2021 and 2024 were retrospectively analysed. Patients were grouped according to Periapical Index (PAI) scores. Systemic immune‐inflammation index (SII), neutrophil‐to‐lymphocyte ratio (NLR), platelet‐to‐lymphocyte ratio (PLR) and platelet inflammatory value (PIV) were calculated from routine blood counts. Severe AP (PAI scores 4–5) was associated with significantly higher SII, NLR, and PIV values (*p* < 0.05). The number of teeth with PAI score 5 correlated positively with SII, NLR and PIV, whereas PLR showed no significant association. Greater severity of AP was associated with elevated systemic inflammatory indices. PIV may provide adjunctive information on systemic inflammatory response in patients with severe AP.

## 1 Introduction

Apical periodontitis (AP) is an inflammatory disease of the periapical tissues that develops as a consequence of persistent microbial infection within the root canal system [1]. Asymptomatic periapical lesions may remain undiagnosed for extended periods, leading to progressive bone destruction and potentially contributing to an increased inflammatory cell burden in the systemic circulation [2].

Periodontal diseases have been widely associated with systemic conditions such as cardiovascular diseases, diabetes mellitus, and respiratory infections [3]. Similarly, dental infections have been suggested to contribute to systemic inflammatory burden, with reported associations between odontogenic infections and conditions such as infective endocarditis as well as cardiovascular diseases [4].

In recent years, haematological inflammatory biomarkers derived from routine complete blood count analysis have gained increasing attention as accessible indicators of systemic inflammatory status. Parameters such as the neutrophil‐to‐lymphocyte ratio (NLR), the platelet‐to‐lymphocyte ratio (PLR), the systemic immune‐inflammation index (SII), and the platelet inflammatory value (PIV) have been shown to reflect inflammatory activity in a variety of systemic diseases [5, 6, 7].

In dentistry, NLR, PLR and SII have been investigated in various clinical conditions, including recurrent aphthous stomatitis, early childhood caries, postoperative edema following orthognathic surgery, prognosis of patients hospitalized due to dental infections, and their potential associations with oral cancers [8, 9, 10, 11, 12]. However, despite the growing interest in the systemic effects of oral infections, data regarding the association between the severity of AP and systemic inflammatory biomarkers in adult patients remain scarce. Moreover, to the best of our knowledge, the potential relationship between AP and PIV has not yet been explored, representing a notable gap in the existing literature.

Therefore, the aim of the present retrospective study was to evaluate the association between the severity of AP and systemic inflammatory markers including SII, NLR, PLR, and PIV in adult patients. It was hypothesized that increased radiographic severity of AP would be associated with elevated systemic inflammatory parameters.

## 2 Materials and Methods

This retrospective study was conducted in accordance with the Declaration of Helsinki, approved by the Ethics Committee of Istanbul Okan University (Meeting No: 188, Decision No: 22, Date: 09 April 2025) and reported in accordance with the STROBE guidelines for observational studies.

Sample size estimation was performed based on the findings reported by Saraç et al. [12] using G*Power software (version 3.1.9.4; Franz Faul, Universität Kiel, Kiel, Germany). Assuming a moderate effect size of 0.40 for differences between three groups, a power of 80% (1–β = 0.80), and a significance level of *α* = 0.05, the minimum required sample size was calculated as 194 patients. The final study population consisted of 206 patients, exceeding the minimum required sample size.

Archived clinical, radiographic, and laboratory data of systemically healthy adult patients who underwent dental treatment under general anaesthesia between 2021 and 2024 were retrospectively evaluated. Patients were considered systemically healthy if they had no history of systemic inflammatory, autoimmune, metabolic, cardiovascular or chronic infectious diseases. To minimize potential confounding effects on systemic inflammatory parameters, current smokers, individuals with obesity (BMI ≥ 30 kg/m^2^), recent acute infections (within 4 weeks), or recent use of antibiotics, corticosteroids or anti‐inflammatory medications were excluded. All patients were treated under general anaesthesia according to institutional clinical protocols. Indications for general anaesthesia were related to treatment complexity, multiple procedures or limited patient cooperation rather than systemic medical conditions.

Peripheral venous blood samples were obtained as part of routine preoperative laboratory assessment according to institutional protocols. In our hospital setting, preoperative blood tests are routinely obtained in the fasting state within 24 h before dental treatment under general anaesthesia. Complete blood count analyses were performed using an automated haematology analyser (Sysmex XN‐1000, Sysmex Corporation, Kobe, Japan) in accordance with standardized laboratory procedures and internal quality control protocols. Reference ranges were as follows: neutrophils (2.0–6.9 × 10^3^/μL), lymphocytes (0.6–3.4 × 10^3^/μL), monocytes (0.0–0.9 × 10^3^/μL), and platelets (150–450 × 10^3^/μL).

Demographic variables including age and sex were recorded for each patient. From the electronic medical records, absolute platelet (PLT), neutrophil (NEUT), lymphocyte (LYMPH), and monocyte counts were obtained from complete blood count analyses routinely requested for all patients as part of the preoperative assessment.

The following systemic inflammatory indices were calculated using absolute cell counts:

$$\text{Systemic immune‐inflammation index}\;\left(\mathrm{SII}\right)=\left(\mathrm{PLT}\times \text{NEUT}\right)/\text{LYMPH}$$

$$\text{Neutrophil‐to‐lymphocyte ratio}\;\left(\mathrm{NLR}\right)=\text{NEUT}/\text{LYMPH}$$

$$\text{Platelet‐to‐lymphocyte ratio}\;\left(\mathrm{PLR}\right)=\mathrm{PLT}/\text{LYMPH}$$

$$\text{Platelet inflammatory value}\;\left(\mathrm{PIV}\right)=\left(\mathrm{PLT}\times \text{NEUT}\times \text{MONO}\right)/\text{LYMPH}$$

For preoperative assessment, intraoral periapical radiographs obtained using the parallel technique as part of routine clinical practice were analysed. All radiographs were evaluated on a digital imaging system under standardized viewing conditions. Periapical lesions were independently evaluated by two experienced endodontists, who calibrated prior to the evaluation using a subset of radiographs not included in the final analysis. Interobserver agreement was assessed using Cohen's kappa coefficient, which demonstrated excellent agreement (*κ* = 0.951, *p* < 0.05). Periapical status was evaluated using the Periapical Index (PAI) scoring system described by Ørstavik et al. [13], which classifies periapical conditions into five radiographic categories: (1) Normal periapical structures, (2) Small changes in bone structure, (3) Structural bone changes with minimal mineral loss, (4) Periodontitis with a well‐defined radiolucent area, (5) Severe periodontitis with exacerbating features.

Only multirooted teeth were included to ensure a more standardized radiographic assessment and to account for the potentially greater inflammatory burden associated with complex root anatomy and furcation involvement. For each tooth, the highest PAI score among all roots was recorded. At the patient level, classification was based on the highest PAI score identified among all evaluated multirooted teeth. Accordingly, patients were stratified into three groups: Group 1 (PAI score 1), Group 2 (PAI scores 2–3), and Group 3 (PAI scores 4–5). Groups 2 and 3 represented increasing severity of periapical pathology, with PAI scores of 4–5 indicating clearly established periapical lesions and scores of 2–3 reflecting less pronounced radiographic changes.

Statistical analyses were performed using IBM SPSS Statistics version 27 (IBM Corp., Armonk, NY, USA). The distribution of continuous variables was assessed using the Kolmogorov–Smirnov test, which showed that the data were not normally distributed. Therefore, non‐parametric statistical methods were preferred for the analyses. Descriptive statistics were presented as minimum, maximum, mean, standard deviation and median values. The Chi‐square test was used to compare the sex distribution among the groups. Differences between the groups were analysed using the Kruskal–Wallis test. When a statistically significant difference was detected in the Kruskal–Wallis test, pairwise comparisons were performed using the Dunn–Bonferroni post hoc test to control for the probability of Type I error resulting from multiple comparisons.

For correlation analysis, the number of teeth with PAI score 5 was used to represent the highest level of periapical inflammatory burden. Correlations between the number of teeth with PAI score 5 and systemic inflammatory markers were evaluated using Spearman's rho correlation analysis. To evaluate the diagnostic performance of systemic inflammatory biomarkers in identifying severe periapical periodontitis, receiver operating characteristic (ROC) curve analysis was performed. Through ROC analysis, the optimal cut‐off values for the biomarkers were determined, along with their corresponding sensitivity and specificity values.

The demographic characteristics of the participants (age and sex distribution) were also evaluated, and no statistically significant differences were found between the groups in terms of these variables. Therefore, these variables were considered unlikely to have a substantial confounding effect on the study results. A *p* < 0.05 was considered statistically significant.

## 3 Results

The study was conducted with a total of 206 patients, whose ages ranged from 16 to 76 years, including 103 females (50%) and 103 males (50%). According to PAI scores, 47 patients (22.8%) were classified as Group 1, 89 patients (43.2%) as Group 2, and 70 patients (34.0%) as Group 3. Age and sex distribution were comparable among the study groups, with no statistically significant differences observed (*p* > 0.05) (Table 1).

**TABLE 1:** Evaluation of the groups in terms of age and sex.

	Group 1 (*n* = 47)	Group 2 (*n* = 89)	Group 3 (*n* = 70)	*p*
Age, mean ± SD (median) (min–max)	40.23 ± 13.61 (40) (20–70)	35.12 ± 13.23 (32) (16–76)	35.93 ± 11.56 (33) (19–68)	0.079^a^
Sex, *n* (%)				
Male	23 (48.9%)	40 (44.9%)	40 (57.1%)	0.307^b^
Female	24 (51.1%)	49 (55.1%)	30 (42.9%)	

^a^ Kruskal‐Wallis test.

^b^ Chi‐square test.

The comparisons of SII, NLR, PLR, and PIV values among the three groups are presented in Table 2. A statistically significant difference was observed among the groups in terms of SII, NLR, and PIV values (*p* = 0.021, 0.002, 0.001). Group 3 exhibited significantly higher SII, NLR, and PIV values than both Group 1 (*p* = 0.012, 0.006, 0.002) and Group 2 (*p* = 0.026, 0.010, 0.021). No significant difference was found between Group 1 and Group 2 (*p* > 0.05). In contrast, no statistically significant difference was found among the groups with respect to PLR values (*p* = 0.472).

**TABLE 2:** Comparison of systemic inflammatory indices (SII, NLR, PLR and PIV) among the groups.

	Group 1 (*n* = 47)	Group 2 (*n* = 89)	Group 3 (*n* = 70)	*p*
	Mean ± SD (Median) (Min–Max)	Mean ± SD (Median) (Min–Max)	Mean ± SD (Median) (Min–Max)	
SII	395.1 ± 175.85 (351.47) (142.49–968.03)	417.36 ± 190.65 (366) (154.05–1081.28)	566.66 ± 434.86 (431.37) (137.35–2318.85)	0.021*
NLR	1.62 ± 0.75 (1.39) (0, 4‐3,63)	1.64 ± 0.63 (1.67) (0.53–3.79)	2.25 ± 1.3 (1.76) (0.63–6.92)	0.002*
PLR	116.96 ± 38.11 (108.06) (48.63–257.14)	115.26 ± 40.31 (113.46) (36.47–247.37)	112.53 ± 50.41 (101.55) (54.07–428.89)	0.472
PIV	241.30 ± 248.50 (184.56) (65.39–1593.4)	246.38 ± 194.86 (219.87) (65.82–1683.95)	473.94 ± 477.74 (244.79) (65.81–2146.67)	0.001*

*Note:* Kruskal Wallis test.

* *p* < 0.05.

**FIGURE 1:**
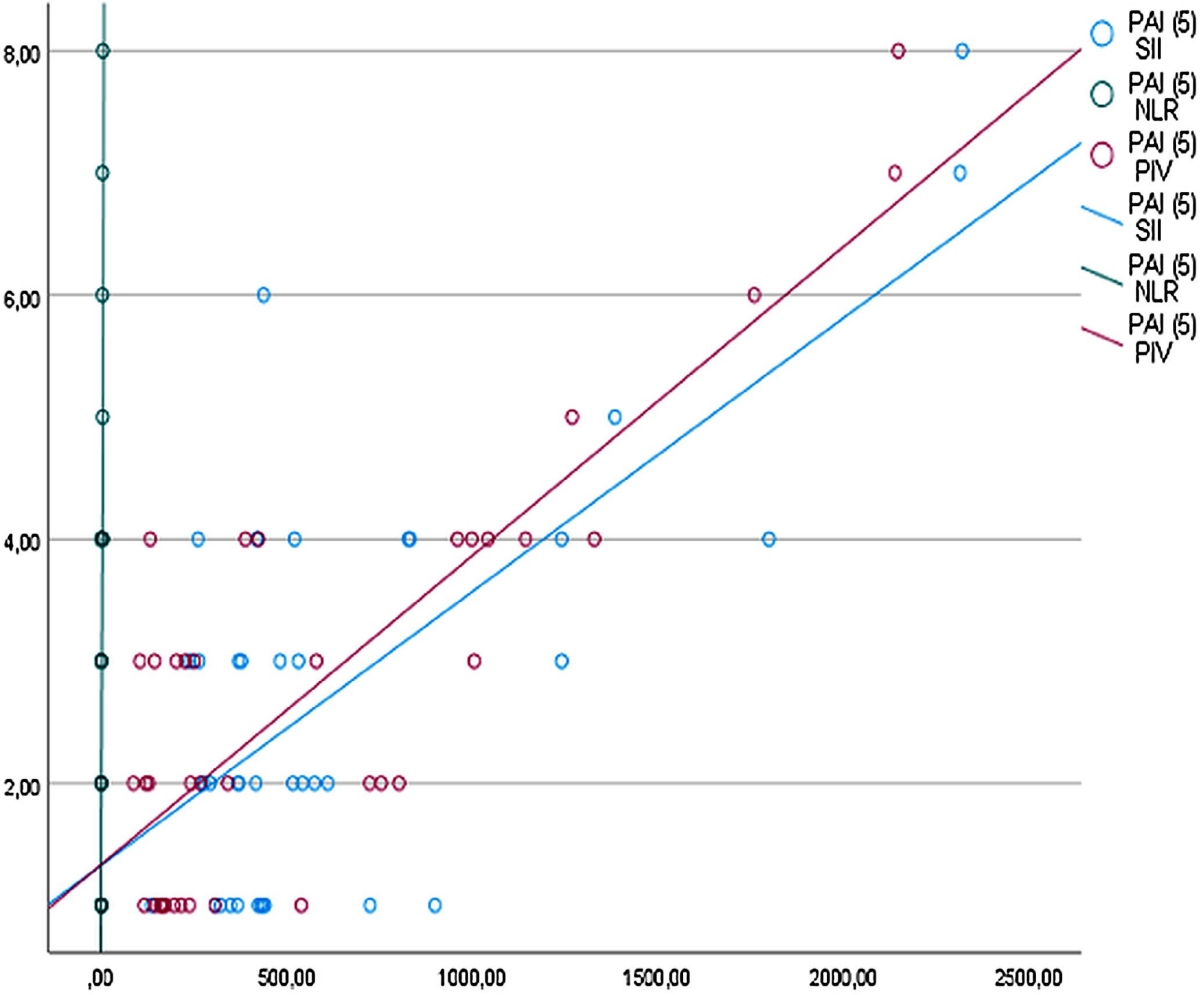
Scatter plots illustrating the correlations between the number of teeth with PAI score 5 and systemic inflammatory indices (SII, NLR and PIV). Linear regression lines are shown for each parameter.

**FIGURE 2:**
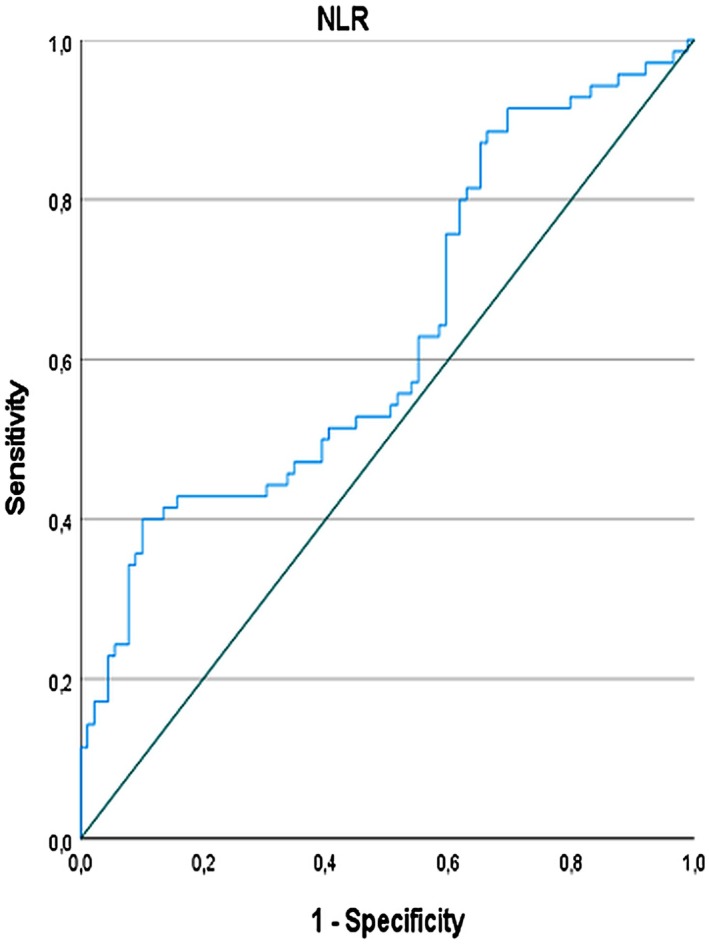
ROC curve of NLR levels for identifiying severe AP. AUC was 0.631 (%95 Cl: 0.551‐0.706).

**FIGURE 3:**
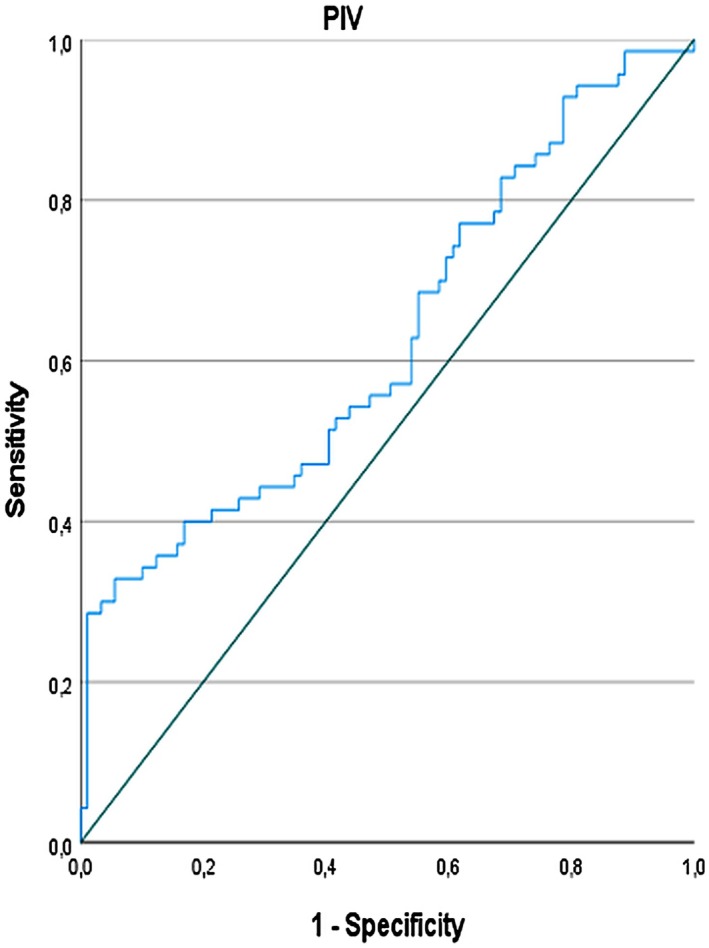
ROC curve of PIV levels for identifiying of severe AP. AUC was 0.627 (%95 Cl: 0.547‐0.702).

**FIGURE 4:**
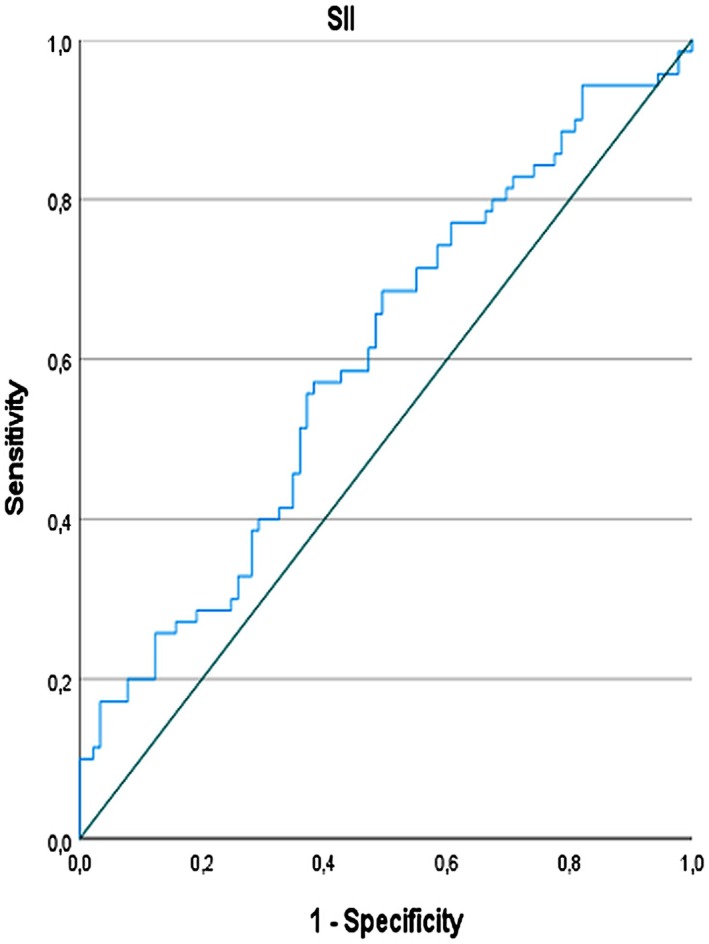
ROC curve of SII levels for identifiying of severe AP. AUC was 0.601 (%95 Cl: 0.520‐0.678).

The correlations between the number of teeth with PAI score 5 and systemic inflammatory markers are summarized in Table 3. The number of teeth with PAI score 5 showed a moderate positive correlation with SII values (*r* = 0.406, *p* = 0.010) and NLR values (*r* = 0.554, *p* = 0.001), as well as a strong positive correlation with PIV values (*r* = 0.610, *p* = 0.001), whereas no significant correlation was observed with PLR values (*p* > 0.05) (Figure 1). ROC curve analysis was performed to evaluate the diagnostic performance of SII, NLR, and PIV in identifying severe AP (Group 3) (Table 4). ROC analysis showed statistically significant discriminatory ability for NLR (AUC = 0.631, *p* = 0.004), PIV (AUC = 0.627, *p* = 0.005), and SII (AUC = 0.601, *p* = 0.026). The optimal cut‐off values were > 2.19 for NLR, > 577 for PIV, and > 366 for SII, with corresponding sensitivity and specificity values shown in Figures 2, 3, 4.

**TABLE 3:** Correlation between the number of teeth with PAI score 5 and SII, NLR, PLR and PIV.

	Number of teeth with PAI score 5	
	*r*	*p*
SII	0.406	0.010*
NLR	0.554	0.001*
PLR	−0.121	0.464
PIV	0.610	0.001*

*Note:* Spearman's rho correlation test.

* *p* < 0.05.

**TABLE 4:** Receiver operating characteristic (ROC) curve analysis with cut‐off values of SII, NLR and PLR for identifying severe AP.

	AUC	SE	95% CI	*p*	Cut off point	Sensitivity	Specificity
NLR	0.631	0.05	0.551–0.706	0.004*	> 2,19	40	88.8
PIV	0.627	0.05	0.547–0.702	0.005*	> 577	28.6	98.9
SII	0.601	0.05	0.520–0.678	0.026*	> 366	68.6	50.6

*Note:* * indicates statistical significance (*p* < 0.05).

## 4 Discussion

NLR is an established marker of chronic systemic inflammation and has demonstrated prognostic value in various inflammatory and chronic disease states [14]. SII, which integrates neutrophil, lymphocyte, and platelet counts, provides a comprehensive measure of systemic inflammatory burden [15]. Accordingly, NLR and SII were selected in the present study to reflect neutrophil‐driven inflammatory activity associated with chronic periapical infection. In addition, platelet‐based indices, including PLR and PIV, were evaluated to assess the contribution of platelet‐related and innate immune pathways.

The present study demonstrated that increased severity of periapical lesions was associated with significantly higher SII, NLR and PIV values, whereas PLR did not show significant differences among groups. In addition, a positive association was observed between the number of teeth with severe periapical lesions (PAI score 5) and these systemic inflammatory indices, suggesting that cumulative periapical disease burden may be reflected in systemic inflammatory status.

AP is a chronic odontogenic infection characterized by persistent microbial stimulation and host inflammatory response [1]. Although often asymptomatic, untreated periapical lesions may act as continuous sources of inflammatory mediators and bacterial by‐products, potentially contributing to low‐grade systemic inflammation [16, 17]. Similar to observations in periodontal diseases [3], the present findings support the concept that chronic endodontic infections may exert systemic effects beyond the local periapical environment.

Among the evaluated biomarkers, SII and NLR increased progressively with higher PAI scores, reflecting enhanced neutrophil activity in response to chronic bacterial challenge. Neutrophils are key effector cells of the innate immune system and represent the primary cellular response to bacterial infections [18, 19]. In contrast, platelet and lymphocyte counts remained comparable between groups, likely reflecting their more limited involvement in the immediate innate response to bacterial pathogens [19, 20, 21]. Platelets are mainly involved in hemostatic processes and tissue repair [20], whereas lymphocytes predominantly participate in adaptive immune mechanisms, particularly those related to viral infections or prolonged immune‐mediated conditions [21]. This differential behaviour highlights the importance of evaluating multiple inflammatory indices rather than relying on a single parameter.

Notably, PIV exhibited the strongest association with severe AP and with the number of teeth presenting PAI score 5. By incorporating neutrophil, platelet, monocyte, and lymphocyte counts, PIV reflects multiple components of the inflammatory and immune response. To the best of our knowledge, this is the first study to evaluate the relationship between AP severity and PIV, suggesting that this index may be particularly sensitive to cumulative inflammatory burden and may provide complementary information to traditional neutrophil‐based indices such as NLR and SII. Also from a clinical perspective, PIV may provide additional insight into the cumulative systemic inflammatory burden in patients presenting with multiple severe periapical lesions.

The present findings are best interpreted in the context of the limited existing literature. To date, the study by Saraç et al. represents the most comparable investigation examining the relationship between periapical pathology severity and systemic inflammatory indices in children with early childhood caries [12]. Despite differences in patient age, both studies demonstrated concordant increases in neutrophil‐driven indices, including NLR and SII, with increasing periapical disease severity. Furthermore, ROC curve analyses in both studies revealed statistically significant but modest discriminatory ability for NLR and SII, with comparable AUC values. Importantly, while Saraç et al. focused on NLR and SII, the present study extended this approach by additionally evaluating PIV, which demonstrated a discriminatory performance comparable to that of NLR and SII. These findings suggest that the systemic inflammatory response is influenced primarily by the severity and chronicity of periapical infection rather than patient age alone.

Several limitations should be acknowledged. The retrospective design precludes causal inference, and the study population consisted of patients treated under general anaesthesia. Although indications for general anaesthesia were related to treatment complexity and behavioural considerations rather than systemic health, this specific clinical setting may limit the generalizability of the findings. Additionally, periapical assessment was based on two‐dimensional radiographs, potentially underestimating lesion extent. Despite these limitations, the study benefits from a sufficiently powered sample size, standardized radiographic assessment and comprehensive evaluation of multiple systemic inflammatory biomarkers.

In conclusion, increasing severity and extend of apical periodontitis were associated with elevated systemic inflammatory indices, particularly SII, NLR and PIV, in systemically healthy adults. These findings support the concept that chronic periapical infections may contribute to systemic inflammatory burden and suggest that next‐generation haematological biomarkers, especially PIV, may serve as useful adjunctive indicators in the assessment of patients with severe AP. From a clinical perspective, timely diagnosis and management of apical periodontitis may be important not only for local infection control but also for minimizing potential systemic inflammatory effects. Further prospective and longitudinal studies are required to clarify the clinical implications of these associations and to evaluate changes in systemic inflammatory status following endodontic treatment.

## Author Contributions

All authors have contributed significantly, and all authors are in agreement with the manuscript.

## Funding

The authors have nothing to report.

## Ethics Statement

This study was approved by Istanbul Okan University Ethics Commitee (Meeting No: 188, Decision No: 22, Date: 09 April 2025).

## Conflicts of Interest

The authors declare no conflicts of interest.

## Data Availability

The data that support the findings of this study are available from the corresponding author upon reasonable request.
